# The transcriptional landscape of mouse beta cells compared to human beta cells reveals notable species differences in long non-coding RNA and protein-coding gene expression

**DOI:** 10.1186/1471-2164-15-620

**Published:** 2014-07-22

**Authors:** Christopher Benner, Talitha van der Meulen, Elena Cacéres, Kristof Tigyi, Cynthia J Donaldson, Mark O Huising

**Affiliations:** Razzavi Newman Integrated Genomics and Bioinformatics Core, The Salk Institute for Biological Studies, 10010 North Torrey Pines Road, La Jolla, CA 92037 USA; Clayton Foundation Laboratories for Peptide Biology, The Salk Institute for Biological Studies, 10010 North Torrey Pines Road, La Jolla, CA 92037 USA; Department of Neurobiology, Physiology & Behavior, University of California, One Shields Avenue, 180 Briggs Hall, Davis, CA 95616 USA

**Keywords:** Beta cell, Alpha cell, Pancreatic islet, Insulin, Glucagon, Ucn3, Crh, Diabetes, lncRNA, RNA-seq, Prolactin receptor, Prlr, Growth hormone receptor, Ghr, Ciliary neurotrophic factor receptor, Cntfr, Islet amyloid polypeptide, Iapp, Amylin, Ets1

## Abstract

**Background:**

Insulin producing beta cell and glucagon producing alpha cells are colocalized in pancreatic islets in an arrangement that facilitates the coordinated release of the two principal hormones that regulate glucose homeostasis and prevent both hypoglycemia and diabetes. However, this intricate organization has also complicated the determination of the cellular source(s) of the expression of genes that are detected in the islet. This reflects a significant gap in our understanding of mouse islet physiology, which reduces the effectiveness by which mice model human islet disease.

**Results:**

To overcome this challenge, we generated a bitransgenic reporter mouse that faithfully labels all beta and alpha cells in mouse islets to enable FACS-based purification and the generation of comprehensive transcriptomes of both populations. This facilitates systematic comparison across thousands of genes between the two major endocrine cell types of the islets of Langerhans whose principal hormones are of cardinal importance for glucose homeostasis. Our data leveraged against similar data for human beta cells reveal a core common beta cell transcriptome of 9900+ genes. Against the backdrop of overall similar beta cell transcriptomes, we describe marked differences in the repertoire of receptors and long non-coding RNAs between mouse and human beta cells.

**Conclusions:**

The comprehensive mouse alpha and beta cell transcriptomes complemented by the comparison of the global (dis)similarities between mouse and human beta cells represent invaluable resources to boost the accuracy by which rodent models offer guidance in finding cures for human diabetes.

**Electronic supplementary material:**

The online version of this article (doi:10.1186/1471-2164-15-620) contains supplementary material, which is available to authorized users.

## Background

Pancreatic beta and alpha cells are clustered together in pancreatic islets to ensure tight coordination of the secretion of insulin and glucagon, whose opposing actions on hepatic glucose metabolism are essential for glucose homeostasis. Yet, despite the functional juxtaposition of insulin and glucagon, pancreatic beta and alpha cells derive from a common progenitor population that during embryonic development is first uniquely defined by the expression of the basic helix-loop-helix transcription factor neurogenin 3 (*Neurog3*) [1]. Under the influence of lineage-specific sets of transcription factors, *Neurog3*+ early endocrine progenitors undergo stepwise differentiation along parallel lineages to develop into mature alpha and beta cells [2–5]. It follows that upon completion of their differentiation and maturation, alpha and beta cells, by virtue of the secretion of their signature hormones glucagon and insulin, have adapted to functionally opposing roles in glucose metabolism. Insulin acts on most peripheral tissues to facilitate the clearance of glucose from the general circulation by inhibiting hepatic gluconeogenesis and promoting hepatic glycogen synthesis and glucose uptake in skeletal muscle and adipocytes [6, 7]. Furthermore, insulin conveys important metabolic feedback to hypothalamic feeding centers regarding the energy state of the body [8]. In contrast, the hepatic actions of glucagon, which include the stimulation of glycogenolysis, provide an essential counter-regulatory mechanism to ensure stable glycemic control [9]. The serious consequences of the absolute insulin deficiency that occur secondary to autoimmune attack of the beta cells in type 1 diabetes as well as the relative insulin deficiency characteristic of type 2 diabetes illustrate the critical importance of insulin. However, the view that hyperglucagonaemia contributes significantly to the progression of both major forms of diabetes has gained acceptance [10]. It follows that a comprehensive understanding of both the alpha and the beta cell and how they regulate glucose homeostasis through the coordination of their activity quite possibly will hold the key to therapeutic interventions aimed at curbing the current diabetes epidemic.

The intimate clustering of beta and alpha cells in pancreatic islets subserves the tight coordination of the secretion of insulin and glucagon, but has long complicated the process of obtaining highly pure populations of alpha and beta cells from isolated islets. To overcome this challenge, we have developed a reporter strain that fluorescently marks all beta cells by the nuclear expression of mCherry under control of the mouse insulin 1 (*Ins1*) promoter and crossed it to a GFP reporter line that labels alpha cells. Bitransgenic offspring of a cross between both lines then enabled the collection of purified populations of alpha and beta cells from the same islets. These tools enabled the first transcriptome-wide comparison of murine alpha and beta cells by RNA-seq, which revealed over 2500 genes that are differentially expressed (p-value < 1 × 10^-7^, false discovery rate (FDR) < 0.1%) between both populations, with insulin the second-most significantly enriched in the beta cell fraction. Next generation sequencing combines the tremendous benefits in sensitivity and dynamic range of techniques traditionally used to assess expression of targeted sets of genes, such as quantitative PCR, with the scale of microarray and the distinct benefit that no a priori sequence information is required, which enables novel transcript discovery. We take advantage of our data to conduct the first transcriptome-wide assessment of the similarities and differences that exist between rodent and human beta cells. These data provide an unprecedented global view of the core beta cell transcriptome that is conserved across species, while simultaneously highlighting marked differences in the expression of receptors and associated long non-coding RNAs (lncRNAs) between beta cells of both species. An unbiased and comprehensive comparison between mouse and human beta cells is an invaluable resource that can facilitate the translation of preclinical findings in rodent models towards therapeutic strategies aimed at alleviating or curing diabetes.

## Results

### Generation and validation of mIns1-H2b-mCherry and S100b-eGFP reporter lines

**Figure 1:**
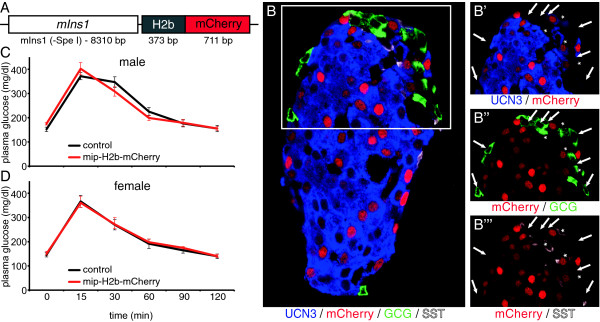
**Generation of a beta cell reporter mouse that faithfully and selectively marks all beta cells.** A fusion of histone-2b (H2b) and monomeric cherry (mCherry) was inserted downstream of the long *Ins1* promoter fragment **(A)** to generate a mIns1-H2b-mCherry beta cell reporter mouse that features nuclear expression of mCherry in all beta cells **(B)**. Male **(C)** and female **(D)** mIns1-H2b-mCherry mice demonstrate normal glucose control as demonstrated by glucose tolerance test compared to wild type littermates.

While available beta cell reporter lines such as the MIP-GFP and MIP-RFP lines [11], facilitate the purification of pure populations of beta cells, reporter expression in these lines is mosaic, which leaves as much as half or more of all beta cells not marked [12]. To obtain a beta cell reporter line that faithfully and selectively labels all beta cells, we generated a transgenic reporter line by cloning an open reading frame encoding a fusion protein between human histone-2b (H2b) and monomeric Cherry (mCherry) downstream of a 8.3 kB mouse insulin 1 promoter (generously gifted by Dr. Mark Magnuson) (Figure 1A). The resulting mIns1-H2b-mCherry transgenic reporter mice demonstrate nuclear mCherry expression in all beta cells (Figure 1B) and are healthy, viable and normoglycemic with no sign of glucose intolerance (Figure 1C, D). To co-label alpha cells, we capitalized on the serendipitous discovery that enhanced green fluorescent protein (eGFP) expression in the S100b-eGFP transgenic reporter line [13] labels alpha cells within the islet (Additional file 1). As there is no detectable expression of endogenous *S100b* transcript in purified S100b + alpha cells, the expression of eGFP in alpha cells is an artifactual, but useful trait that enables the purification of alpha cells by FACS.

### Whole transcriptome analysis of highly enriched mouse beta and alpha cells

**Figure 2:**
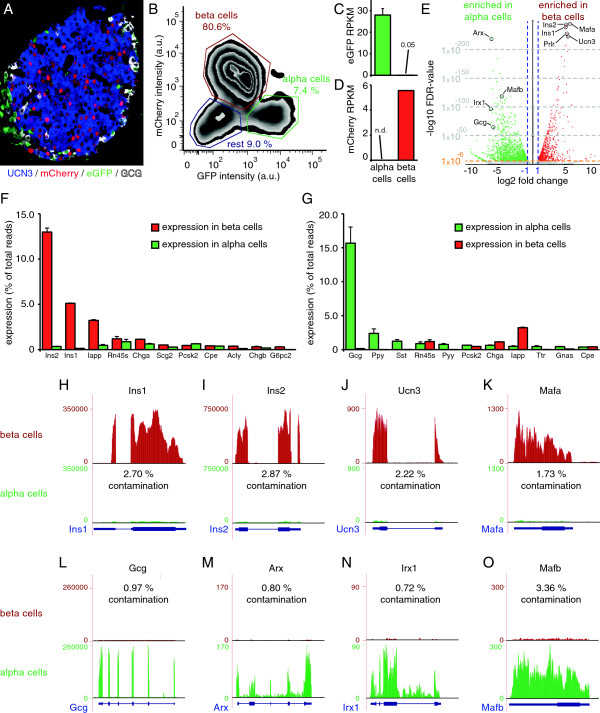
**Validation of comprehensive transcriptomes of mouse beta and alpha cells.** Bitransgenic offspring of mIns1-H2b-mCherry x S100b-eGFP bitransgenic reporter mice **(A)** enable the FACS purification of pure populations of beta and alpha cells **(B)**. Expression of eGFP is negligible in mCherry + beta cells **(C)**, and mCherry expression in eGFP + alpha cells is not detected **(D)**. A volcano plot highlights key alpha and beta cell-enriched genes **(E)**. The most abundantly expressed genes in beta **(F)** and alpha **(G)** cells are expressed by the fraction of total reads that maps to each gene and compared to expression in the opposite cell type. Expression of the beta cell markers *Ins1* **(H)**, *Ins2* **(I)**, *Ucn3* **(J)** and *Mafa* **(K)** in alpha cells measures on average less than 2.39% of expression in beta cells. Conversely, expression of the alpha cell markers *Gcg* **(L)**, *Arx* **(M)**, *Irx1* **(N)** and *Mafb* **(O)** in mouse beta cells is on average even lower. See also Additional files 1, 2 and 3.

Islets isolated from two replicate groups of bitransgenic offspring of a cross between the mIns1-H2b-mCherry and S100b-eGFP reporter lines (Figure 2A) enable the simultaneous purification of alpha and beta cells by FACS (Figure 2B). We detected not a single read in our eGFP + alpha cell fractions that maps to mCherry, and only three reads that map to eGFP in our mCherry + beta cell fractions, underscoring the quality of our FACS purification strategy (Figure 2C, D). Furthermore, while the expression of the endogenous Ins1 gene measures in at an average RPKM (reads per kilobase of exon model per million mapped reads) [14] value of approximately 230,000 (Additional file 2), the use of the 10 kb mouse *Ins1* promoter to drive H2b-mCherry transcription in the beta cells results in mCherry RPKM values of only slightly over 5. This relatively low mRNA expression despite the use of one of the strongest promoters in the beta cell context partially explains the relatively dim mCherry signal in the nuclei of beta cells and may have fortuitously contributed to the fact that our mIns1-H2b-mCherry beta cell reporter mice are healthy and do not display or develop any beta cell defects that would precipitate diabetes (Figure 1C, D).

A comprehensive comparison of the transcriptomes of alpha and beta cells revealed 2547 genes that were differentially expressed between beta and alpha cells. A total of 1075 genes were significantly (p-value < 1 × 10^-7^, FDR < 0.1%) enriched in beta cells. The Insulin 2 (*Ins2*) and *Ins1* genes are the second- and third most significantly enriched genes in beta cells compared to alpha cells, after the beta cell-specific transcription factor *Mafa* (Figure 2E; Additional file 2), which serves as a potent confirmation of the high purity of the beta cell population obtained by the use of our mIns1-H2b-mCherry reporter mouse. Other genes that were highly significantly enriched in mouse beta cells are genes encoding the prolactin receptor (*Prlr*) and the peptide hormone Urocortin3 (*Ucn3*) (Figure 2E). Conversely, 1472 genes were significantly (p-value < 1 × 10^-7^, FDR < 0.1%) enriched in the alpha cell fraction, including the mouse alpha cell-specific transcription factors *Arx*, *Irx1*, *Mafb*, as well as glucagon (*Gcg*) (Additional file 3). It is no surprise that *Ins2* and *Ins1* are the most abundantly detected transcripts in beta cells, comprising on average 13.0% and 5.1%, respectively, of all mapped reads in the beta cell libraries (Figure 2F). In the alpha cell-enriched libraries, *Gcg* is responsible for on average 15.7% of all mapped reads. Expression of other islet hormones, including pancreatic polypeptide (*Ppy*), somatostatin (*Sst*), peptide YY (*Pyy*) and islet amyloid polypeptide (*Iapp*), is between 6.5 and 33-fold lower than the expression of *Gcg*, which confirms the successful enrichment of alpha cells (Figure 2G). While the abundance of *Ins2*/*Ins1* and *Gcg* transcripts is expected in beta- and alpha cell-enriched libraries, respectively, the fraction of reads that maps to insulin is lower than for human beta cells, where upwards of 38% of all mapped reads in FACS-purified beta cells were assigned to *INS* [15, 16]. As a further validation of our FACS purification strategy, expression of known mouse beta cell-specific genes in the alpha cell fraction, including *Ins1*, *Ins2*, *Ucn3* [17, 18] and *Mafa* [19], was on average less than 2.39% of the expression detected in beta cells (Figure 2H-K). Conversely, transcripts encoding the alpha cell-transcription factors *Arx1*, *Irx1* and *Mafb* and *Gcg*, which are all highly significantly enriched in alpha cells (Additional file 3), are detectable in the beta cell fraction at on average less than 1.49% of their expression in alpha cells (Figure 2L-O). We compared our alpha and beta cell transcriptomes to a previous study that contrasted FACS-purified MIP-GPF + beta cells to transcriptomes of intact islets [20]. Their effort was somewhat limited by the aforementioned mosaicism of the MIP-GFP reporter line [11], which reduced the contrast between the transcriptomes of MIP-GFP + beta cells and total islets [20]. This manifested as the unremarkable enrichment of key beta cell markers including *Ins2*, *Ins1*, *Mafa* and others in this study compared to our work (Additional file 4).

### Functional validation of alpha and beta cell transcriptomes

**Figure 3:**
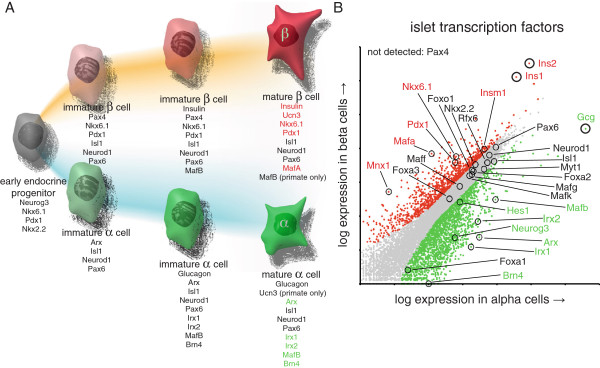
**Comparison of mouse beta and alpha cell transcriptomes based on functional annotation.** Establishment and maintenance of alpha and beta cell identity is regulated by a complex interplay of transcription factors **(A)**, whose expression pattern is accurately reflected by our mouse alpha and beta cell transcriptomes. Actual transcription factor expression in beta and alpha cells is represented by a dot plot where each dot represents an individual gene **(B)**. Genes that are significantly enriched (p < 1 × 10^-7^) in beta or alpha cells are highlighted in red and green, respectively, with genes enriched with p < 1 × 10^-50^ displayed in bold for emphasis. *Ins1*, *Ins2*, and *Gcg* are marked for reference. See also Additional files 5 and 6.

While global pathway analysis of beta cell-enriched genes by DAVID [21] reveals significant association with keywords linked to aspects of beta cell function and disease (Additional file 5), generic functional categories can lack the resolution to discriminate between relatively similar cell types such as alpha and beta cells. We therefore assessed the expression of the transcription factors that determine alpha and beta cell identity, often in a mutually repressive fashion (Figure 3A) [4, 22]. The abundant expression of the transcription factors *Pdx1*, *Nkx6.1*, *Mafa* and *Mnx1* in mature beta cells (Figure 3B) is in close agreement with several reports that demonstrate their expression to be necessary for mature beta cell identity and function [4, 23–25]. *Mafb*, *Arx*, *Irx1* and *Irx2* are significantly enriched in mouse alpha cells. Interestingly, many transcription factors that are required for proper islet development and function, such as *Pax6*, *Neurod1*, *Isl1*, *Rfx6*, *Nkx2.2* , *Myt1*, *Foxo1*, and *Foxa2* are robustly expressed in both alpha and beta cell fractions and are not significantly, or only modestly enriched in either population. *Neurog3* is required for the formation of the endocrine pancreas [1] and remains detectable at relatively low levels (Figure 3B), but is depleted from beta cells (Figure 3B) in contrast to reports that attribute *Neurog3* expression in adult islets to mature beta cells [26–28]. *Pax4* is important to repress the alpha cell-determining transcription factor *Arx* and facilitate differentiation along the beta and delta lineage [22, 29], but is undetectable in mature mouse beta cells (Figure 3B) [30]. We similarly compared the relative enrichment of key genes involved in glucose sensing, stimulus secretion coupling and insulin exocytosis between alpha and beta cells and found that the facilitated glucose transporter *Slc2a2* (a.k.a. *Glut2)*, glucose-6-phosphate convertase 2 (*G6pc2)*, proprotein convertase subtilisin/kexin type 1 (*Pcsk1*), Ero 1-like beta (*Ero1lb*), and synaptotagmin-like 4 (*Sytl4*, a.k.a. granuphilin) were highly significantly enriched in beta cells, while genes encoding the ATP-sensitive potassium channel and voltage-gated calcium channels were equally abundantly expressed in alpha and beta cells (Additional file 6).

### Differences between mouse and human islet cells

**Figure 4:**
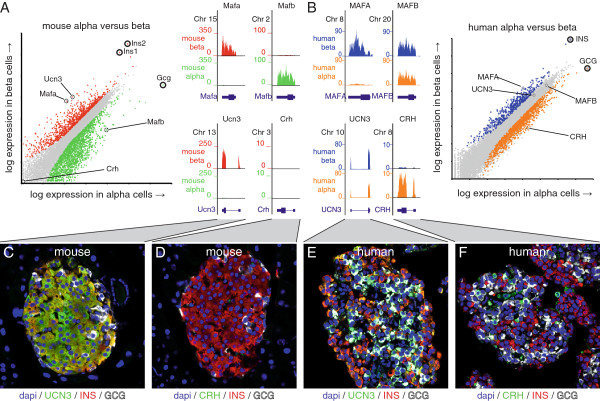
**Validation of known differences in gene expression between mouse and human alpha and beta cells.** We assessed the expression of two sets of genes, *Mafa*/*Mafb* and *Ucn3*/*Crh*, that markedly differ in distribution between alpha and beta cells in mouse **(A)** and human **(B)** islets. *Mafa* is restricted to beta cells of both species, while *Mafb* is selectively expressed in mouse alpha cells **(A)**, but expressed in human beta cells as well **(B)**. *Ucn3* is highly selective for mouse beta cells while *Crh* is detected in neither mouse islet population **(A, C, D)**. By contrast, *UCN3* expression is a common feature of human alpha and beta cells, while *CRH* is enriched in human alpha cells **(B, E, F)**. Human RNA-seq data are from [16]. See also Additional file 7 for single channel images.

Mice are widely used as models for islet disease, despite marked differences between rodent and primate islet architecture and innervation [31–34]. The full transcriptomes of mouse beta and alpha cells enable a comprehensive comparison of both the similarities and differences in gene expression between mouse and human alpha and beta cells. To validate the approach, we compared the expression of genes with known species differences between mouse and human alpha and beta cells. *Mafa* and *Mafb* are selectively expressed in adult mouse alpha and beta cells, respectively (Figure 4A) [19, 35]. In contrast, while *Mafa* expression is similarly restricted to human beta cells, *Mafb* expression is retained in human beta cells and therefore not enriched in either human islet population (Figure 4B) [36, 37]. As a second example of species differences between mouse and human islets, *Ucn3* is highly selectively expressed in mouse beta cells [17, 18] (Figure 2E; Figure 4A, C; Additional file 7) but is a common feature of human alpha and beta cells [17]. This is reflected by robust expression of human *UCN3* but lack of enrichment in either human islet cell type (Figure 4B, E; Additional file 7). The gene encoding the related peptide hormone corticotropin-releasing hormone (*Crh*), best known as the principal hypothalamic initiator of the stress response, is not detectable in mouse islets (Figure 4A, D) but is highly enriched in human alpha cells (Figure 4B, F; Additional file 7), likely explaining the endogenous insulinotropic activity in human islets that is blocked by the selective CRH_1_ receptor blocker antalarmin [12].

### Comprehensive comparison of mouse and human beta cell transcriptomes

**Figure 5:**
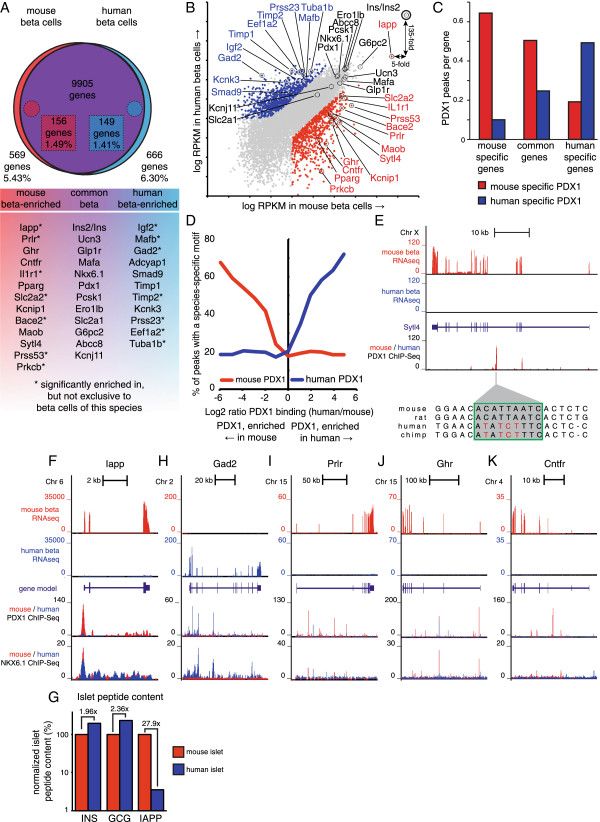
**Comparison of the mouse and human beta cell transcriptomes.** The unbiased comparison of mouse and human transcriptomes reveals a common core of over 9900 genes that are expressed at an RPKM value > 1 in beta cells of both species **(A)**. A total of 725 genes (569 unique and 156 enriched) was robustly (>10-fold) and significantly (p < 1 × 10^-7^) higher expressed in mouse beta cells (**B**, red) while 815 genes (666 unique and 149 enriched) were by the same criteria significantly higher expressed in human beta cells (**B**, blue). Genes enriched in mouse beta cells were co-enriched for mouse-specific PDX1 binding and *vice versa* **(C)**. Moreover, enrichment of PDX1 ChIP-Seq peaks in either species is associated with species-specific PDX1 binding motifs **(D)**, as illustrated for *Sytl4* **(E)**. *Iapp* transcript levels in purified beta cells **(F)** and islet IAPP peptide content **(G)** are markedly reduced in human compared to mouse. Additional browser plots demonstrate notable examples of significantly enriched or selectively expressed genes in either species including *Gad2* **(H)**, *Prlr* **(I)**, *Ghr* **(J)** and *Cntfr* **(K)**. Human RNA-seq data are from [16], ChIP-Seq data are from [38, 49]. See also Additional files 8, 9 and 10.

Having thus validated our approach, we proceeded with a global comparison of the genes enriched in mouse and human beta cells using a comparable set of transcriptomes for human FACS-purified beta cells [16]. One has to be very careful when conducting cross-species comparisons of next-generation sequencing libraries that are generated by different investigators using different protocols. To minimize the potential caveats associated with such a comparison, we built in several safeguards in our analysis pipeline. We reanalyzed the raw human sequence files in the same manner as our mouse data and used transcriptome data from beta cells of both species expressed as RPKM to allow direct, unbiased comparison. This yielded a total of 9905 genes that demonstrated RPKM expression values > 1 in both mouse and human beta cells and thus constitute the common beta cell transcriptome (Additional file 8). This extensive list includes many of the key genes that define beta cell identity such as *Ins2*/*INS*, *Ucn3*, *Mafa*, *Nkx6.1*, *Pdx1*, *Ero1lb*, *Glp1r*, *G6pc2*, and the ATP-sensitive potasssium channel subunits *Abcc8* and *Kcnj11* (Figure 5A). To identify the most striking species differences between mouse and human beta cells, we focused only on the genes that demonstrated both significant enrichment (p-value < 1 × 10^-7^, FDR < 0.1%) and over 10-fold difference in beta cells of one species over the other. This approach identified 725 genes that were either uniquely expressed (human beta RPKM < 1, n = 569) or significantly enriched (human beta RPKM > 1, n = 156) in mouse beta cells (Figure 5A, B; Additional file 9). Conversely, we identified 815 genes as either uniquely expressed (mouse beta RPKM < 1, n = 666) or significantly enriched (mouse beta RPKM > 1, n = 149) in human beta cells (Figure 5A, B; Additional file 9). Significant enrichment in gene expression in one species correlated with the presence of Pdx1 ChIP-Seq peaks specific to that species (Figure 5C) [38], which in turn correlated strongly with the presence of species-specific PDX1 binding motifs (Figure 5D). This suggests that differences in the mouse and human PDX1 cistrome contribute to the differential expression of genes between mouse and human beta cells, as is illustrated by the *Sytl4* gene, which is uniquely expressed in mouse beta cells and is associated with a strong mouse-specific PDX1 ChIP-Seq peak that overlies a consensus *Pdx1* binding motif in the mouse that is absent from human (Figure 5E).

The small subset of 156 genes that is significantly (p < 1 × 10^-7^, FDR < 0.1%) and robustly (>10-fold) enriched in mouse beta cells, but still detectable in human beta cells, constitutes less than 1.5% of the common beta cell transcriptome (Figure 5A). This cluster includes *Iapp*, which is more than 30-fold reduced in mouse over human beta cells (Figure 5B, F), perhaps in adaptation to the amyloid properties of human but not mouse IAPP that contribute to the onset and progression of type 2 diabetes [39]. The strongly disparate relative expression of the gene encoding IAPP in mouse and human islets is in contrast to other hormones such as *INS*/*Ins2* and *Ucn3*, which are expressed at similar levels between mouse and human beta cells (Figure 5A, B). We further validated the relatively low expression of *Iapp* message by demonstrating that IAPP peptide content is reduced almost 28-fold in human islets compared to mouse, while INS and GCG content is similar in islets from both species (Figure 5G; Additional file 10). A similarly small set of 149 genes that is detectably expressed in beta cells of both species, but robustly and significantly enriched in human beta cells includes *Gad2* (Figure 5B, H), which is a major auto-antigen in human type 1 diabetes [40, 41].

### Notable differences in cell surface receptors between human and mouse beta cells

Several of the most notable gene expression differences between mouse and human beta cells concern cell surface receptors of the class I helical cytokine receptor family [42]. *Prlr* has long been implicated in beta cell mass expansion in adaptation to the increased metabolic demand of pregnancy in rodents in response to PRL or placental lactogens [43, 44]. Indeed, *Prlr* is abundantly and selectively expressed in mouse beta cells (Figures 2E, 5A, B; Additional file 2). In contrast, while *Prlr* is detectable in FACS-purified human beta cells and is associated with a human PDX1 ChIP-Seq signature (Figure 5I), it is expressed at approximately 15-fold lower RPKM values than mouse beta cells (Figure 5B, Additional file 9) and is significantly depleted compared to co-purified human non-beta cells (not shown). Similarly, related receptor genes for growth hormone (*Ghr*) (Figure 5J) and ciliary neurotrophic factor (*Cntfr*) (Figure 5K) are robustly expressed by mouse beta cells, but are virtually undetectable in human beta cells.

### Novel transcript discovery

**Figure 6:**
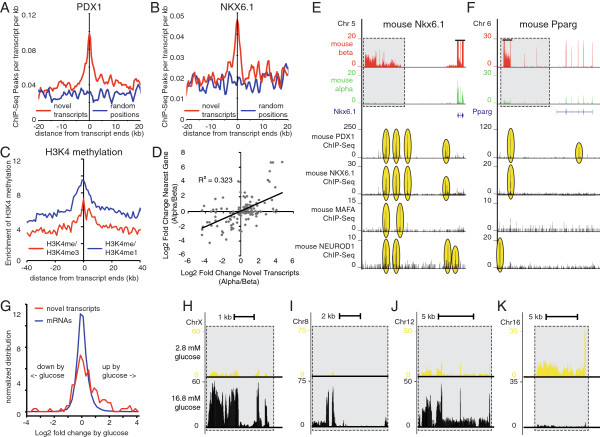
**The mouse alpha and beta cell transcriptomes reveal unique beta cell-specific lncRNAs.** Mouse islet lncRNAs demonstrate significant enrichment for binding by the beta cell-specific transcription factors PDX1 **(A)** and NKX6.1 **(B)**, are enriched for H3K4me1 and H3K4me3 markers of transcriptionally active loci **(C)** and are co-regulated with their nearest protein-coding neighbor **(D)**. This is illustrated by lncRNAs adjacent to *Nkx6.1* **(E)** and *Pparg* **(F)**, which are co-enriched in mouse beta cells with these protein-coding genes and strongly associate with binding of beta cell-specific transcription factors. Overnight stimulation of mouse islets with 16.8 mM glucose strongly regulates a subset of lncRNAs in comparison to protein-coding transcripts **(G)** as illustrated by lncRNAs that are significantly up regulated by glucose at chromosome X **(H)**, 8 **(I)** and 12 **(J)** or down regulated by glucose at chromosome 16 **(K)**. ChIP-Seq and H3K4 data are from [38, 45, 48, 69]. Numbers inside the grey boxes refer to lncRNA coordinates and correspond to Additional file 11.

**Figure 7:**
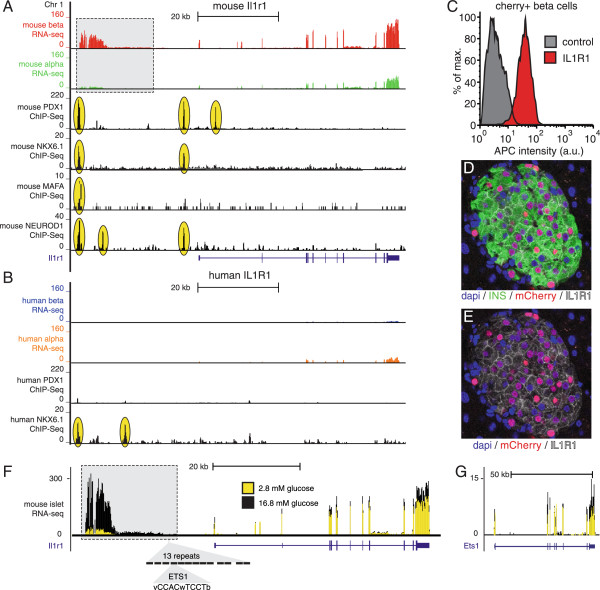
**The** ***Il1r1*** **locus is uniquely regulated in mouse beta cells.** *Il1r1* is highly enriched in mouse beta cells when compared to both mouse alpha cells **(A)** and human beta cells **(B)**. This expression pattern is mimicked by a beta cell-specific lncRNA upstream of the *Il1r1* transcription start in the mouse but not human locus. Both the *Il1r1* transcription start site and the upstream lncRNA in mouse are associated with strong transcription factor binding for PDX1, NKX6.1, MAFA and NEUROD1, while there is only limited NKX6.1 binding at the human locus **(B)**. Cell surface expression of IL1R1/CD121a on mIns1-H2b-mCherry + beta cells is confirmed by FACS **(C)** and immunofluorescence **(D, E)**. Both *Il1r1* and its associated lncRNA are significantly up regulated by overnight stimulation of mouse islets with 16.8 mM glucose **(F)**. The *Il1r1* lncRNA features 13 repeats of the ETS1 consensus binding site within a 300 bp stretch of DNA (MCAST p value = 0.0009), which may offer an explanation for the strong glucose regulation of *Il1r1* and its associated lncRNA, as *Ets1* expression is significantly increased by elevated glucose **(G)**. Human RNA-seq data are from [16], ChIP-Seq data are from [38, 48, 69]. Numbers inside the grey box refer to lncRNA coordinates and correspond to Additional file 11.

We leveraged our combined alpha and beta RNA-seq expression data for novel islet transcript discovery in the mouse islet. Our initial list of novel transcripts included 73% of the lncRNAs identified in purified mouse beta cells [20] and 54% of the lncRNAs identified from total mouse islets [15]. We formulated a stringent set of criteria (see Methods) to generate a set of 145 novel lncRNA transcripts. Of these, 18 are positioned within the introns of known protein-coding genes, while the remaining 127 are situated in intergenic regions, the majority of which were not identified in previous studies of mouse islet lncRNAs [15, 20] (Additional file 11). The transcript ends for these novel islet transcripts were significantly enriched for transcription factor binding by PDX1 (Figure 6A), NKX6.1 (Figure 6B), MAFA and NEUROD1 (not shown) compared to random positions. They also were enriched for histone H3 lysine 4 (H3K4) mono-methyl and tri-methyl signatures that mark transcriptionally active loci (Figure 6C) [45]. These observations underscore that these novel lncRNAs are transcriptionally active and suggest transcriptional regulation by beta cell specific transcription factors. As lncRNAs are emerging as an additional layer in the regulation of gene expression of neighboring protein-coding genes, we compared the relative enrichment of lncRNAs in beta cells with the enrichment of their nearest protein-coding gene. This revealed that the ratio of expression in mouse beta and alpha cells is correlated between these novel transcripts and their nearest protein-coding gene (Figure 6D). This general phenomenon has also been noted for human islet-specific lncRNAs [15] and is illustrated by novel lncRNAs associated with *Il1r1* (Figure 7), *Nkx6.1* (Figure 6E) and *Pparg* (Figure 6F), which are all co-enriched in mouse beta over alpha cells in concert with their closest protein-coding gene. To test whether these novel beta cell lncRNAs are subject to transcriptional regulation, we stimulated primary mouse islets overnight with 16.8 mM glucose and compared their expression to non-stimulated control islets from the same pool. Several of our set of 145 lncRNAs were among the most significantly up regulated (Figure 6H-J) or down regulated (Figure 6K) by glucose of all coding and non-coding transcripts.

### The interleukin-1 receptor locus in beta cells

The interleukin-1 receptor type 1 (*Il1r1*/CD121a) locus aptly recapitulates all aspects of our study. *Il1r1* is the most abundantly expressed cell surface receptor on mouse beta cells by a considerable margin with an RPKM value > 200. Expression of *Il1r1* is significantly enriched in mouse beta over alpha cells (p < 1.9 × 10^-15^) and the mouse *Il1r1* promoter is associated with strong transcription factor binding by the beta cell transcription factors PDX1, NKX6.1 and NEUROD1 (Figure 7A), validating the beta cell as the source of *Il1r1* expression. In sharp contrast, *IL1R1* expression in human islet populations, while detectable, is significantly lower in human beta cells in comparison to both mouse beta cells (Figure 5A, B) and human alpha cells (Figure 7A, B). The high levels of *Il1r1* message in mouse beta cells translate into abundant cell surface expression of IL1R1/CD121a on mCherry + beta cells as measured by FACS (Figure 6C) and confocal microscopy (Figure 7D, E). An additional notable feature unique to the mouse *Il1r1* locus is a lncRNA positioned about 30 kb upstream of the *Il1r1* transcription start site. This lncRNA demonstrates strong beta cell enrichment similar to the adjacent *Il1r1* transcript and is associated with significant transcription factor binding by PDX1, NKX6.1, MAFA and NEUROD1 (Figure 7A). The corresponding region of the human *IL1R1* locus shows no human PDX1 binding or evidence for the expression of a similar lncRNA in islet cells (Figure 7B). Moreover, expression of this mouse-specific lncRNA upstream of *Il1r1* is the most significantly up regulated of all transcripts, coding and non-coding, following overnight stimulation of mouse islets with glucose (Figure 7F; Additional file 11). A repeat of 13 consensus binding sites for the transcription factor V-Ets Avian Erythroblastosis Virus E26 Oncogene Homolog 1 (ETS1) (Figure 6F) that is absent from the human *IL1R1* locus may contribute to both the expression of this lncRNA by mouse but not human beta cells and the robust glucose-induced up regulation of its expression, as *Ets1* expression is significantly up regulated by glucose (Figure 7G).

## Discussion

To ensure tight coordination of the secretion of insulin and glucagon, pancreatic beta and alpha cells are intimately colocalized in the pancreas, which has long complicated the process of obtaining highly pure populations of alpha and beta cells from isolated islets. We successfully overcame this problem by generating a mIns1-H2b-mCherry beta cell reporter line that enabled us to achieve over 98% beta cell purity, which is markedly higher than previous studies that used antibody-based strategies to purify beta cells from dissociated human islet suspensions [15, 16, 46]. The fact that both mouse insulin genes rank as the second and third most significantly enriched genes in mouse beta over alpha cells - trailing only the key beta cell-specific transcription factor *Mafa* - underscores our high quality of beta cell enrichment. Further confirmation that our transcriptomes faithfully recapitulate known expression patterns in alpha and beta cells follows from the fact that expression of key alpha and beta cell transcription factors as well key genes involved in glucose sensing, stimulus secretion coupling and insulin exocytosis generally adheres closely to the literature [2–5]. It is noteworthy that many additional genes involved in glycolysis, membrane depolarization, calcium entry and insulin exocytosis are not, or only modestly enriched in mouse beta over alpha cells, suggestive of similarities in the triggering of hormone release by either cell type as was recently reported [47].

Importantly, our transcriptome data now offer the opportunity for the systematic comparison of gene expression by mouse and human beta cells. We identified a core set of 9906 genes that are shared between mouse and human beta cells. It is no surprise that this set contains many of the key genes required for the stimulus-appropriate release of insulin such as *G6pc2*, *Ero1lb*, *Pcsk1* and *Glp1r*. We also uncovered significant transcriptome differences between mouse and human beta cells that are accompanied with species-specific enrichment of beta cell-specific transcription factors, and sometimes associated with species-specific lncRNAs. We minimized the potential caveats that can stem from differences in islet isolation, dissociation, FACS sorting and library preparation between laboratories by focusing only on robustly (10-fold enrichment) and significantly differentially expressed (p < 1 × 10^-7^, FDR < 0.1) genes. Further validation of transcriptome differences between mouse and human beta cell transcriptomes was obtained by demonstrating that species-specific enrichment of gene expression correlates well with species-specific enrichment of ChIP signatures for the beta cell specific transcription factors Pdx1 [38] and Nkx6.1 [48, 49]. Among the more striking examples are *Prlr*, *Ghr* and *Cntfr*, three related receptors that respond to structurally similar ligands [42], that are abundantly and selectively expressed by mouse beta cells, with only nominal expression in human islets. Co-stimulation of CNTF and EGF promotes acinar to beta cell transdifferentiation in diabetic mice [50] and GH and PRL are potent inducers of mouse beta cell proliferation [51] and implicated in the expansion of maternal beta cell mass during pregnancy in mice [43, 44]. However, efforts to use PRL or GH to induce human beta cell proliferation *in vitro* have been largely unsuccessful [52, 53] and expansion of human beta cell mass during pregnancy is modest compared to mouse and driven by neogenesis instead of beta cell proliferation [54]. While there are clearly differences between mouse and human beta cells that prevent the latter from entering the cell cycle [55], the lack of *GHR* and relative absence of *PRLR* are not conducive to robust responses to their potential mitogenic ligands.

The most abundantly expressed cell surface receptor in mouse beta cells, measured by gene expression, is *Il1r1*, which responds to the pro-inflammatory cytokines IL-1α and IL-1β. Importantly, the human islet is not devoid of *IL1R1* transcript, in line with a body of literature that established that mouse and human beta cells respond to sustained IL-1β stimulation with a reduction in function and an increase in apoptosis [56–58]. The robustly elevated *Il1r1* expression in mouse islets could betray a more prominent role for locally produced IL-1β in beta cell glucotoxicity in mouse islets and potentially render mouse beta cells more sensitive to IL-1β-mediated pro-inflammatory insults. This observation is important, as local inflammation in the islet precipitated by obese conditions is thought to contribute to beta cell failure and exacerbate diabetes [59].

Several of the genes that displayed markedly higher expression in mouse over human beta cells, such as *Il1r1* and *Pparg*, were flanked by mouse-specific novel lncRNAs. We therefore leveraged our transcriptome data to identify 145 novel lncRNAs that are enriched for significant transcription factor binding of key beta cell transcription factors and are co-regulated with the nearest protein-coding gene in line with previous observations [15, 60, 61]. As lncRNAs are not bound by the constraints of protein-coding genes, they tend to display less sequence conservation and stability over longer phylogenetic distances [62]. Indeed, comparison of the set of 145 mouse lncRNAs with human [15] revealed evidence for a human orthologous lncRNA for only two. It appears that the repertoire of islet lncRNAs is dynamic and has changed considerably since the divergence of primates and rodents, in line with the general notion that the lncRNA repertoire across vertebrates is dynamic, even between closely related species [60, 63, 64]. Their generally high plasticity and poor sequence conservation notwithstanding, lncRNAs are emerging as an important and novel transcriptional regulatory mechanism that is likely to significantly impact beta cell fate and function. Indeed, we observed that in response to stimulation with glucose, the quintessential beta cell trigger, quite a few lncRNAs were up- or down regulated by 10-fold or more. The arduous task of attributing function to the beta cell-enriched lncRNAs that have emerged from ours and similar studies [15, 20] is now underway.

## Conclusion

Rodents are commonly used to study the islets of Langerhans, with the ultimate goal of improving the outlook of diabetic patients and despite considerable differences in islet architecture and innervation between rodent and primate islets. Our comprehensive beta and alpha cell transcriptomes will greatly enhance our understanding of normal islet physiology and yield significant new leads to direct the behavior of its two primary endocrine constituents. Importantly, our data leveraged against recently published transcriptomes for FACS-purified human islet populations will now facilitate routine cross comparison between mouse and human alpha and beta cell transcriptomes. This is an invaluable resource for all with an interest in islet physiology and disease that stands to improve the translatability of rodent studies [65] by ensuring that potential therapeutic targets identified by preclinical experiments on rodents are similarly expressed by human beta cells.

## Methods

### Generation of a mIns1-H2b-mCherry beta cell reporter line

Animals were maintained on a 12-h light/12-h dark cycle with free access to water and standard rodent how. DNA encoding a fusion between human histone-2b and monomeric Cherry (H2b-mCherry) was ligated in the Spe I site of the mouse insulin 1 promoter (pBS.MIP1(-SpeI); generously gifted by Dr. Mark Magnuson). The resulting mIns1-H2b-mCherry reporter construct (Figure 1A) was used for pronuclear injection by the Salk transgenic core, leading to two founder lines. One founder line demonstrated bright nuclear expression of mCherry in only a small minority of beta cells and was discarded. The other founder line was determined to have the transgenic cassette inserted at two separate loci, one of which was silent and was bred out. No fertility or viability issues were noted and reporter expression was only observed in the pancreas. Although no effect of transgene copy number was observed, only hemizygous offspring were used. The mIns1-H2b-mCherry reporter line was crossed to S100b-eGFP mice (The Jackson Laboratory, Bar Harbor, ME; strain 005621) [13]. Glucose tolerance tests were carried out as previously described [12]. All procedures were approved by the Salk Institute for Biological Studies Institutional Animal Care and Use Committee.

### Islet isolation and FACS sorting

Islet isolation was carried out as previously described [12]. Islets from mIns1-H2b-mCherry × S100b-eGFP bitransgenic animals were pooled by sex in two replicate groups of 10 or 11 animals. In preparation for sorting, islets were hand-picked into a 15 ml conical tube and allowed to sediment before excess media was aspirated. Islets were dissociated at 37°C by adding 0.25% trypsin-EDTA (Invitrogen) aided by regular but gentle mechanical dissociation with a p200 pipette. Dissociated islet cells were washed in HBSS containing 10% FBS and sorted at the Salk Institute Flow Cytometry core on a FACS Vantage SE DiVa (Becton-Dickinson, Franklin Lakes, NJ) using 488 and 568 excitation lines for eGFP and mCherry, respectively. Sorted cells were collected directly in Trizol. Flow cytometric staining for Il1r1/CD121a-APC (Biolegend, San Diego, CA) was conducted on dissociated mCherry + beta cells at 5 microgram/ml in 100 microliter HBSS + 10% FBS. For glucose stimulation, islets from 3 month old male C57Bl6 mice (Harlan, Indianapolis, IN) were cultured overnight in RPMI containing 11 mM glucose, before glucose deprivation for 2 hours in RPMI containing 2.8 mM glucose followed by 12 h incubation with 2.8 mM or 16.8 mM glucose. One hundred islets were used per replicate.

### RNA isolation and library prep

RNA was isolated from Trizol by a chloroform extraction, assisted by phase lock tubes. RNA was precipitated by isopropanol and cleaned up over an RNEasy microcolumn (Qiagen, Valencia, CA) per the manufacturer's instructions, taking great care to avoid carry over of ethanol following the column washes. Following elution in 30 microliter elution buffer, RNA quality was verified by BioAnalyzer (Agilent, Santa Clara, CA). Indexed sequencing libraries were constructed using the TruSeq RNA sample Prep Kit v2 (Illumina Inc. San Diego, CA) and sequenced at 50 cycles, single read on an Illumina HiSeq 2000 platform.

### RNA-Seq and ChIP-Seq analysis

Sequencing reads from two beta cell populations and two alpha cell populations were mapped using STAR [66] to the mouse genome version mm9 (NCBI build 37). Over 30 million reads were sequenced for each library with over 83% of sequenced reads aligning uniquely (>93% alignment overall). Bedtools [67] was used to create count tables of the sorted bam files using reads aligning to RefSeq defined exons. DESeq [68] was used for statistical comparison. See Additional file 12 for a count table with all genes in our alpha and beta transcriptomes. Raw RNA-seq sequence files of human beta and non-beta cells [16] and ChIP-Seq data for mouse and human PDX1 [38], mouse and human NKX6.1 [48, 49], and mouse MAFA and NEUROD1 [69], were remapped using STAR (RNA-seq) or bowtie2 (ChIP-Seq) to their respective genomes (hg19, mm9). Normalized genome browser tracks were prepared using HOMER (http://homer.salk.edu) [70] and uploaded into the University of California Santa Cruz genome browser to generate browser plots. ChIP-Seq peaks were determined using HOMER. Individual read alignments, transcripts, and ChIP-Seq peak positions for each human sequencing experiment were converted to the mouse genome using the UCSC liftOver tool. Homologene was used to map gene identifiers between mouse and human. To discover species-specific PDX1 peaks, human PDX1 ChIP-Seq read alignments were converted to the mm9 genome. Peaks were found for both species with respect to the mm9 genome and differentially bound peaks were defined by peak regions containing 4-fold more reads in one species relative to the other. Instances of the PDX1 motif were found using HOMER. Peak positions were converted between mm9 and hg19 genomes using the liftOver tool and scanned independently to identify species-specific motifs. The MEME Suite package MCAST [71] was used for the identification of statistically significant consensus binding sites for the four transcription factors found to be differentially-regulated by glucose (*Ets1*, *Pax6*, *Pdx1*, *Myc*). The full mouse transcriptome was scanned using default parameters. The consensus site motifs used were taken from the JASPAR Database [72].

#### Novel transcript discovery

To identify novel lncRNA, RNA-Seq libraries from all replicates of alpha and beta cells were combined into a single meta-experiment to maximize sensitivity. Two approaches where used to identify transcripts. First, Cufflinks [73] was used with default settings to identify transcripts de novo. Second, the findPeaks program in homer was used with “-style histone” and “-minDist 1500” to identify regions of continuous high read density that Cufflinks missed. This approach yielded 71,730 predicted transcripts, which were used to confirm the absence or presence of transcripts predicted by other studies. Because many of the 71,730 transcripts found by HOMER and Cufflinks were short fragments of RNA found within introns or immediately upstream and downstream of known transcripts, we adopted stringent filtering criteria to identify novel, high-quality lncRNAs. Novel transcripts must (1) not overlap with any known RefSeq or UCSC gene exon, (2) have a total transcript length greater than 3 kb, (3) expression in either alpha or beta cell experiment > 1 RPKM. (4) and not overlap with known rRNA loci. We also excluded transcripts with a PhastCon score > 0.4 which likely reflect mapping artifacts to pseudogenes instead of actual novel transcripts.

### Islet peptide content determination

Islets were sonicated in 300 μl of KREBS ringer buffer, then centrifuged 10 min at 8000 K. The supernatant was transferred to a fresh tube. Stock samples were stored at -20 C. For each assay the samples were diluted in the assay buffer supplied with each individual kit. Human and mouse insulin, glucagon and IAPP islet peptide content were measured with commercially available RIA or ELISA kits (EMD Millipore, Billerica, MA) per the manufacturer's instructions. As cross-reactivity for mouse IAPP had not been determined by EMD Millipore, a separate rat/mouse IAPP (American Peptide Company, Sunnyvale, CA) standard curve was run in equivalent doses and shown to be superimposable with the human IAPP curve supplied with the ELISA, confirming 100% cross-reactivity with mouse IAPP (Additional file 10). Mouse and human islet IAPP samples were each quantified according to their homologous standard curve.

### Immunofluorescence

Immunofluorescence was carried out as previously described [17] using commercial antisera against glucagon (guinea pig anti-glucagon at 1:7000; EMD Millipore, Billerica MA), somatostatin (sheep anti-somatostatin at 1:1000; American Research Products Inc., Waltham MA), insulin (chicken anti-insulin at 1:1000; Abcam) and IL1r1/CD121a (armenian hamster anti-mouse Il1r1/CD121a-APC at 1:100; Biolegend, San Diego, CA). Rabbit antisera against UCN3 (#7218, 1:2000) and CRH (rc70, 1:5000) were generated in house. Secondary antibodies were raised in donkey against each of these host species and were obtained from Jackson Immuno Research Laboratories (West Grove, PA) conjugated to Alexa Fluor-405, -488, -649 or Cy3 and used at 1:600. Dapi was applied as a nuclear stain at 1 microgram/ml and slides were embedded with Prolong Gold antifade reagent (Life Technologies, Carlsbad, CA). All imaging was carried out on Zeiss LSM780 confocal microscopes at The Waitt Advanced Biophotonics Center Core facility of the Salk Institute.

### Human islets

Human donor islets were obtained through the Integrated Islet Distribution Program (IIDP) and declared exempt from approval by the Institutional Review Board of the Salk Institute for Biological Studies.

## Authors’ information

CB is the director of the Razzavi Newman Integrated Genomics and Bioinformatics Core at the Salk Institute for Biological Studies and is an expert bio-informatician, who wrote Homer (Hypergeometric Optimization of Motif Enrichment), a suite of tools for Motif Discovery and next-generation sequence analysis. MOH is a Staff Scientist at the Salk Institute's Clayton Foundation Laboratories for Peptide Biology and will continue to grow his research program at the University of California, Davis in the fall of 2014. His group studies the role of Corticotropin Releasing Hormone (CRH) and the related peptide hormone Urocortin3 in the pancreas. The direct actions of these and other peptide hormones on the pancreas add a novel layer of complexity to the intricate network of signaling molecules that in concert governs beta cell mass and endocrine output of the pancreas.

## Electronic supplementary material

Additional file 1: **Is a figure validating the expression of eGFP in alpha cells of the S100b-eGFP reporter mouse.** (PDF 888 KB)

Additional file 2: **Is a table listing the 100 most significantly enriched genes in mouse beta cells.** (XLSX 48 KB)

Additional file 3: **Is a table listing the 100 most significantly enriched genes in mouse alpha cells.** (XLSX 46 KB)

Additional file 4: **Is a side-by-side comparison of our alpha and beta cell transcriptomes with the beta cell transcriptomes derived from MIP-GFP + beta cells** [20]**.** (PDF 277 KB)

Additional file 5: **Is a table listing the most significantly enriched functional categories in the beta and alpha cell fractions.** (XLSX 43 KB)

Additional file 6: **Is a figure highlighting the differential expression of genes important for glucose uptake and glycolysis, stimulus secretion coupling and insulin exocytosis in mouse beta and alpha cells.** (PDF 536 KB)

Additional file 7: **Is a figure containing single channel images for the immuno-fluorescent panels of Figure** 4 **.** (PDF 2 MB)

Additional file 8: **Is a table listing the genes comprising the common beta cell transcriptome as defined by expression > 1 RPKM in beta cells of both species.** (XLSX 815 KB)

Additional file 9: **Is a table listing genes that are significantly enriched or uniquely expression in mouse or human beta cells.** (XLSX 214 KB)

Additional file 10: **Is a side-by-side comparison of a standard curve of mouse and human IAPP as measured by ELISA, demonstrating 100% cross-reactivity.** (PDF 121 KB)

Additional file 11: **Is a table listing novel transcripts discovered in purified mouse beta and alpha cells and the regulation of their expression by glucose.** (XLSX 90 KB)

Additional file 12: **Is a count table with all genes in our alpha and beta transcriptomes.** (XLSX 4 MB)

## References

[1] Gradwohl G, Dierich A, LeMeur M, Guillemot F (2000). Neurogenin3 is required for the development of the four endocrine cell lineages of the pancreas. Proc Natl Acad Sci U S A.

[2] Arda HE, Benitez CM, Kim SK (2013). Gene regulatory networks governing pancreas development. Dev Cell.

[3] Bramswig NC, Kaestner KH (2011). Transcriptional regulation of alpha-cell differentiation. Diabetes Obes Metab.

[4] Schaffer AE, Taylor BL, Benthuysen JR, Liu J, Thorel F, Yuan W, Jiao Y, Kaestner KH, Herrera PL, Magnuson MA, May CL, Sander M (2013). Nkx6.1 controls a gene regulatory network required for establishing and maintaining pancreatic Beta cell identity. PLoS Genet.

[5] Ben-Othman N, Courtney M, Vieira A, Pfeifer A, Druelle N, Gjernes E, Faurite B, Avolio F, Collombat P (2013). From pancreatic islet formation to beta-cell regeneration. Diabetes Res Clin Pract.

[6] Lin HV, Accili D (2011). Hormonal regulation of hepatic glucose production in health and disease. Cell Metab.

[7] Leto D, Saltiel AR (2012). Regulation of glucose transport by insulin: traffic control of GLUT4. Nat Rev Mol Cell Biol.

[8] Schwartz MW, Seeley RJ, Tschop MH, Woods SC, Morton GJ, Myers MG, D'Alessio D (2013). Cooperation between brain and islet in glucose homeostasis and diabetes. Nature.

[9] Ramnanan CJ, Edgerton DS, Kraft G, Cherrington AD (2011). Physiologic action of glucagon on liver glucose metabolism. Diabetes Obes Metab.

[10] Unger RH, Cherrington AD (2012). Glucagonocentric restructuring of diabetes: a pathophysiologic and therapeutic makeover. J Clin Invest.

[11] Hara M, Wang X, Kawamura T, Bindokas VP, Dizon RF, Alcoser SY, Magnuson MA, Bell GI (2003). Transgenic mice with green fluorescent protein-labeled pancreatic beta -cells. Am J Physiol Endocrinol Metab.

[12] Huising MO, van der Meulen T, Vaughan JM, Matsumoto M, Donaldson CJ, Park H, Billestrup N, Vale WW (2010). CRFR1 is expressed on pancreatic beta cells, promotes beta cell proliferation, and potentiates insulin secretion in a glucose-dependent manner. Proc Natl Acad Sci U S A.

[13] Zuo Y, Lubischer JL, Kang H, Tian L, Mikesh M, Marks A, Scofield VL, Maika S, Newman C, Krieg P, Thompson WJ (2004). Fluorescent proteins expressed in mouse transgenic lines mark subsets of glia, neurons, macrophages, and dendritic cells for vital examination. J Neurosci.

[14] Mortazavi A, Williams BA, McCue K, Schaeffer L, Wold B (2008). Mapping and quantifying mammalian transcriptomes by RNA-Seq. Nat Methods.

[15] Moran I, Akerman I, van de Bunt M, Xie R, Benazra M, Nammo T, Arnes L, Nakic N, Garcia-Hurtado J, Rodriguez-Segui S, Pasquali L, Sauty-Colace C, Beucher A, Scharfmann R, van Arensbergen J, Johnson PR, Berry A, Lee C, Harkins T, Gmyr V, Pattou F, Kerr-Conte J, Piemonti L, Berney T, Hanley N, Gloyn AL, Sussel L, Langman L, Brayman KL, Sander M (2012). Human beta cell transcriptome analysis uncovers lncRNAs that are tissue-specific, dynamically regulated, and abnormally expressed in type 2 diabetes. Cell Metab.

[16] Nica AC, Ongen H, Irminger JC, Bosco D, Berney T, Antonarakis SE, Halban PA, Dermitzakis ET (2013). Cell-type, allelic, and genetic signatures in the human pancreatic beta cell transcriptome. Genome Res.

[17] van der Meulen T, Xie R, Kelly OG, Vale WW, Sander M, Huising MO (2012). Urocortin 3 marks mature human primary and embryonic stem cell-derived pancreatic alpha and beta cells. PLoS One.

[18] Li C, Chen P, Vaughan J, Blount A, Chen A, Jamieson PM, Rivier J, Smith MS, Vale W (2003). Urocortin III is expressed in pancreatic beta-cells and stimulates insulin and glucagon secretion. Endocrinology.

[19] Matsuoka TA, Artner I, Henderson E, Means A, Sander M, Stein R (2004). The MafA transcription factor appears to be responsible for tissue-specific expression of insulin. Proc Natl Acad Sci U S A.

[20] Ku GM, Kim H, Vaughn IW, Hangauer MJ, Myung Oh C, German MS, McManus MT (2012). Research resource: RNA-Seq reveals unique features of the pancreatic beta-cell transcriptome. Mol Endocrinol.

[21] da Huang W, Sherman BT, Lempicki RA (2009). Bioinformatics enrichment tools: paths toward the comprehensive functional analysis of large gene lists. Nucleic Acids Res.

[22] Collombat P, Mansouri A, Hecksher-Sorensen J, Serup P, Krull J, Gradwohl G, Gruss P (2003). Opposing actions of Arx and Pax4 in endocrine pancreas development. Genes Dev.

[23] Holland AM, Gonez LJ, Naselli G, Macdonald RJ, Harrison LC (2005). Conditional expression demonstrates the role of the homeodomain transcription factor Pdx1 in maintenance and regeneration of beta-cells in the adult pancreas. Diabetes.

[24] Harrison KA, Thaler J, Pfaff SL, Gu H, Kehrl JH (1999). Pancreas dorsal lobe agenesis and abnormal islets of Langerhans in Hlxb9-deficient mice. Nat Genet.

[25] Guo S, Dai C, Guo M, Taylor B, Harmon JS, Sander M, Robertson RP, Powers AC, Stein R (2013). Inactivation of specific beta cell transcription factors in type 2 diabetes. J Clin Invest.

[26] Shimajiri Y, Kosaka Y, Scheel DW, Lynn FC, Kishimoto N, Wang J, Zhao S, German MS (2011). A mouse model for monitoring islet cell genesis and developing therapies for diabetes. Dis Models Mech.

[27] Wang S, Jensen JN, Seymour PA, Hsu W, Dor Y, Sander M, Magnuson MA, Serup P, Gu G (2009). Sustained Neurog3 expression in hormone-expressing islet cells is required for endocrine maturation and function. Proc Natl Acad Sci U S A.

[28] Dror V, Nguyen V, Walia P, Kalynyak TB, Hill JA, Johnson JD (2007). Notch signalling suppresses apoptosis in adult human and mouse pancreatic islet cells. Diabetologia.

[29] Sosa-Pineda B, Chowdhury K, Torres M, Oliver G, Gruss P (1997). The Pax4 gene is essential for differentiation of insulin-producing beta cells in the mammalian pancreas. Nature.

[30] Smith SB, Ee HC, Conners JR, German MS (1999). Paired-homeodomain transcription factor PAX4 acts as a transcriptional repressor in early pancreatic development. Mol Cell Biol.

[31] Cabrera O, Berman DM, Kenyon NS, Ricordi C, Berggren PO, Caicedo A (2006). The unique cytoarchitecture of human pancreatic islets has implications for islet cell function. Proc Natl Acad Sci U S A.

[32] Brissova M, Fowler MJ, Nicholson WE, Chu A, Hirshberg B, Harlan DM, Powers AC (2005). Assessment of human pancreatic islet architecture and composition by laser scanning confocal microscopy. J Histochem Cytochem.

[33] Kim A, Miller K, Jo J, Kilimnik G, Wojcik P, Hara M (2009). Islet architecture: A comparative study. Islets.

[34] Rodriguez-Diaz R, Abdulreda MH, Formoso AL, Gans I, Ricordi C, Berggren PO, Caicedo A (2011). Innervation patterns of autonomic axons in the human endocrine pancreas. Cell Metab.

[35] Artner I, Le Lay J, Hang Y, Elghazi L, Schisler JC, Henderson E, Sosa-Pineda B, Stein R (2006). MafB: an activator of the glucagon gene expressed in developing islet alpha- and beta-cells. Diabetes.

[36] Riedel MJ, Asadi A, Wang R, Ao Z, Warnock GL, Kieffer TJ (2012). Immunohistochemical characterisation of cells co-producing insulin and glucagon in the developing human pancreas. Diabetologia.

[37] Dai C, Brissova M, Hang Y, Thompson C, Poffenberger G, Shostak A, Chen Z, Stein R, Powers AC (2012). Islet-enriched gene expression and glucose-induced insulin secretion in human and mouse islets. Diabetologia.

[38] Khoo C, Yang J, Weinrott SA, Kaestner KH, Naji A, Schug J, Stoffers DA (2012). Research resource: the pdx1 cistrome of pancreatic islets. Mol Endocrinol.

[39] Westermark P, Andersson A, Westermark GT (2011). Islet amyloid polypeptide, islet amyloid, and diabetes mellitus. Physiol Rev.

[40] van Belle TL, Coppieters KT, von Herrath MG (2011). Type 1 diabetes: etiology, immunology, and therapeutic strategies. Physiol Rev.

[41] Jayasimhan A, Mansour KP, Slattery RM (2014). Advances in our understanding of the pathophysiology of Type 1 diabetes: lessons from the NOD mouse. Clin Sci.

[42] Huising MO, Kruiswijk CP, Flik G (2006). Phylogeny and evolution of class-I helical cytokines. J Endocrinol.

[43] Kim H, Toyofuku Y, Lynn FC, Chak E, Uchida T, Mizukami H, Fujitani Y, Kawamori R, Miyatsuka T, Kosaka Y, Yang K, Honig G, van der Hart M, Kishimoto N, Wang J, Yagihashi S, Tecott LH, Watada H, German MS (2010). Serotonin regulates pancreatic beta cell mass during pregnancy. Nat Med.

[44] Schraenen A, Lemaire K, de Faudeur G, Hendrickx N, Granvik M, Van Lommel L, Mallet J, Vodjdani G, Gilon P, Binart N, in't Veld P, Schuit F (2010). Placental lactogens induce serotonin biosynthesis in a subset of mouse beta cells during pregnancy. Diabetologia.

[45] Hoffman BG, Robertson G, Zavaglia B, Beach M, Cullum R, Lee S, Soukhatcheva G, Li L, Wederell ED, Thiessen N, Bilenky M, Cezard T, Tam A, Kamoh B, Birol I, Dai D, Zhao Y, Hirst M, Verchere CB, Helgason CD, Marra MA, Jones SJ, Hoodless PA (2010). Locus co-occupancy, nucleosome positioning, and H3K4me1 regulate the functionality of FOXA2-, HNF4A-, and PDX1-bound loci in islets and liver. Genome Res.

[46] Bramswig NC, Everett LJ, Schug J, Dorrell C, Liu C, Luo Y, Streeter PR, Naji A, Grompe M, Kaestner KH (2013). Epigenomic plasticity enables human pancreatic alpha to beta cell reprogramming. J Clin Invest.

[47] Zhang Q, Ramracheya R, Lahmann C, Tarasov A, Bengtsson M, Braha O, Braun M, Brereton M, Collins S, Galvanovskis J, Gonzalez A, Groschner LN, Rorsman NJ, Salehi A, Travers ME, Walker JN, Gloyn AL, Gribble F, Johnson PR, Reimann F, Ashcroft FM, Rorsman P (2013). Role of KATP channels in glucose-regulated glucagon secretion and impaired counterregulation in type 2 diabetes. Cell Metab.

[48] Taylor BL, Liu FF, Sander M (2013). Nkx6.1 is essential for maintaining the functional state of pancreatic beta cells. Cell reports.

[49] Pasquali L, Gaulton KJ, Rodriguez-Segui SA, Mularoni L, Miguel-Escalada I, Akerman I, Tena JJ, Moran I, Gomez-Marin C, van de Bunt M, Ponsa-Cobas J, Castro N, Nammo T, Cebola I, Garcia-Hurtado J, Maestro MA, Pattou F, Piemonti L, Berney T, Gloyn AL, Ravassard P, Skarmeta JL, Muller F, McCarthy MI, Ferrer J (2014). Pancreatic islet enhancer clusters enriched in type 2 diabetes risk-associated variants. Nat Genet.

[50] Baeyens L, Lemper M, Leuckx G, De Groef S, Bonfanti P, Stange G, Shemer R, Nord C, Scheel DW, Pan FC, Ahlgren U, Gu G, Stoffers DA, Dor Y, Ferrer J, Gradwohl G, Wright CV, Van de Casteele M, German MS, Bouwens L, Heimberg H (2014). Transient cytokine treatment induces acinar cell reprogramming and regenerates functional beta cell mass in diabetic mice. Nat Biotechnol.

[51] Nielsen JH, Svensson C, Galsgaard ED, Moldrup A, Billestrup N (1999). Beta cell proliferation and growth factors. J Mol Med (Berl).

[52] Yamamoto T, Mita A, Ricordi C, Messinger S, Miki A, Sakuma Y, Timoneri F, Barker S, Fornoni A, Molano RD, Inverardi L, Pileggi A, Ichii H (2010). Prolactin supplementation to culture medium improves beta-cell survival. Transplantation.

[53] Parnaud G, Bosco D, Berney T, Pattou F, Kerr-Conte J, Donath MY, Bruun C, Mandrup-Poulsen T, Billestrup N, Halban PA (2008). Proliferation of sorted human and rat beta cells. Diabetologia.

[54] Butler AE, Cao-Minh L, Galasso R, Rizza RA, Corradin A, Cobelli C, Butler PC (2010). Adaptive changes in pancreatic beta cell fractional area and beta cell turnover in human pregnancy. Diabetologia.

[55] Fiaschi-Taesch NM, Kleinberger JW, Salim FG, Troxell R, Wills R, Tanwir M, Casinelli G, Cox AE, Takane KK, Scott DK, Stewart AF (2013). Human pancreatic beta-cell G1/S molecule cell cycle atlas. Diabetes.

[56] Maedler K, Sergeev P, Ris F, Oberholzer J, Joller-Jemelka HI, Spinas GA, Kaiser N, Halban PA, Donath MY (2002). Glucose-induced beta cell production of IL-1beta contributes to glucotoxicity in human pancreatic islets. J Clin Invest.

[57] Bendtzen K, Mandrup-Poulsen T, Nerup J, Nielsen JH, Dinarello CA, Svenson M (1986). Cytotoxicity of human pI 7 interleukin-1 for pancreatic islets of Langerhans. Science.

[58] Donath MY, Storling J, Maedler K, Mandrup-Poulsen T (2003). Inflammatory mediators and islet beta-cell failure: a link between type 1 and type 2 diabetes. J Mol Med.

[59] Donath MY, Dalmas E, Sauter NS, Boni-Schnetzler M (2013). Inflammation in obesity and diabetes: islet dysfunction and therapeutic opportunity. Cell Metab.

[60] Cabili MN, Trapnell C, Goff L, Koziol M, Tazon-Vega B, Regev A, Rinn JL (2011). Integrative annotation of human large intergenic noncoding RNAs reveals global properties and specific subclasses. Genes Dev.

[61] Orom UA, Shiekhattar R (2013). Long noncoding RNAs usher in a new era in the biology of enhancers. Cell.

[62] Mercer TR, Dinger ME, Mattick JS (2009). Long non-coding RNAs: insights into functions. Nat Rev Genet.

[63] Necsulea A, Soumillon M, Warnefors M, Liechti A, Daish T, Zeller U, Baker JC, Grutzner F, Kaessmann H (2014). The evolution of lncRNA repertoires and expression patterns in tetrapods. Nature.

[64] Kutter C, Watt S, Stefflova K, Wilson MD, Goncalves A, Ponting CP, Odom DT, Marques AC (2012). Rapid turnover of long noncoding RNAs and the evolution of gene expression. PLoS Genet.

[65] Couzin-Frankel J (2013). When mice mislead. Science.

[66] Dobin A, Davis CA, Schlesinger F, Drenkow J, Zaleski C, Jha S, Batut P, Chaisson M, Gingeras TR (2013). STAR: ultrafast universal RNA-seq aligner. Bioinformatics.

[67] Quinlan AR, Hall IM (2010). BEDTools: a flexible suite of utilities for comparing genomic features. Bioinformatics.

[68] Anders S, Huber W (2010). Differential expression analysis for sequence count data. Genome Biol.

[69] Tennant BR, Robertson AG, Kramer M, Li L, Zhang X, Beach M, Thiessen N, Chiu R, Mungall K, Whiting CJ, Sabatini PV, Kim A, Gottardo R, Marra MA, Lynn FC, Jones SJ, Hoodless PA, Hoffman BG (2013). Identification and analysis of murine pancreatic islet enhancers. Diabetologia.

[70] Heinz S, Benner C, Spann N, Bertolino E, Lin YC, Laslo P, Cheng JX, Murre C, Singh H, Glass CK (2010). Simple combinations of lineage-determining transcription factors prime cis-regulatory elements required for macrophage and B cell identities. Mol Cell.

[71] Bailey TL, Noble WS (2003). Searching for statistically significant regulatory modules. Bioinformatics.

[72] Wasserman WW, Sandelin A (2004). Applied bioinformatics for the identification of regulatory elements. Nat Rev Genet.

[73] Trapnell C, Williams BA, Pertea G, Mortazavi A, Kwan G, van Baren MJ, Salzberg SL, Wold BJ, Pachter L (2010). Transcript assembly and quantification by RNA-Seq reveals unannotated transcripts and isoform switching during cell differentiation. Nat Biotechnol.

