# Supplementary material for: The transcriptional landscape of mouse beta cells compared to human beta cells reveals notable species differences in long non-coding RNA and protein-coding gene expression
Source: BMC Genomics. 2014 Jul 22;15(1):620. doi: 10.1186/1471-2164-15-620 (PMC4124169; doi:10.1186/1471-2164-15-620)
Supplement: Supplementary file 1 — Additional file 1: Is a figure validating the expression of eGFP in alpha cells of the S100b-eGFP reporter mouse. (PDF 888 KB) [file 12864_2014_6324_MOESM1_ESM.pdf]

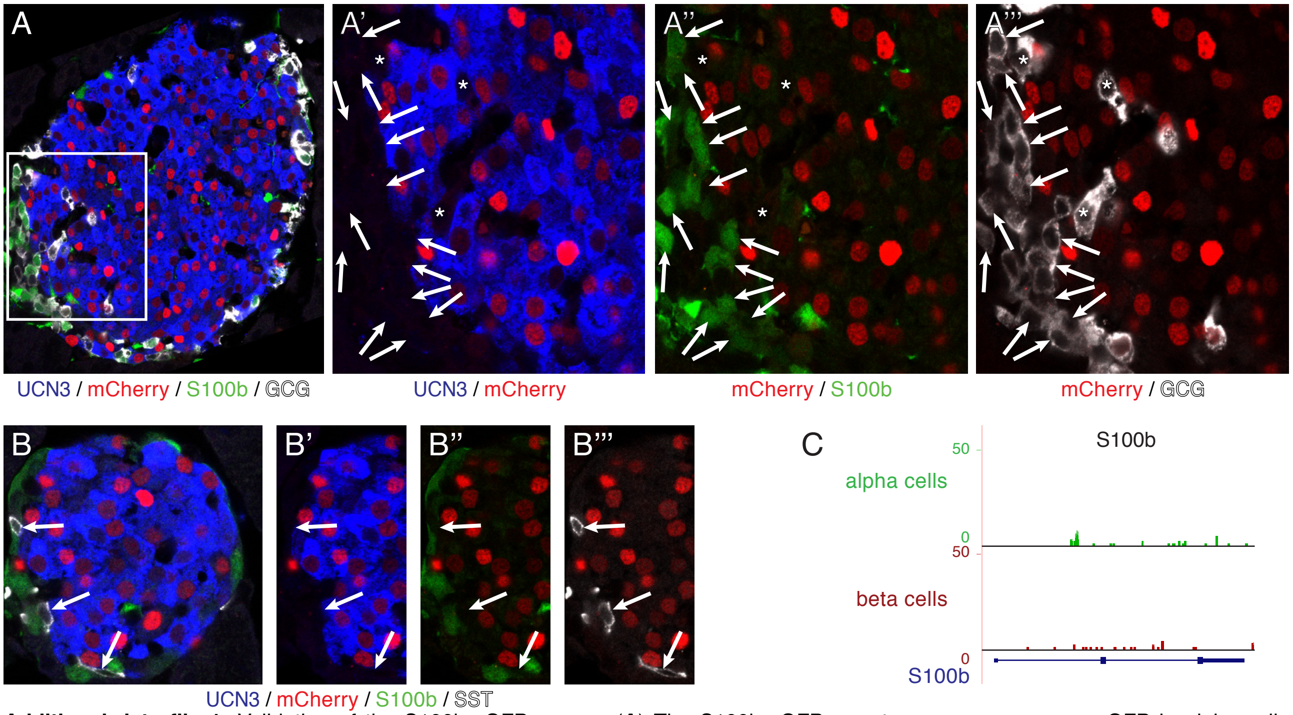

Additional data file 1: Validation of the S100b-eGFP mouse. (**A**) The S100b-eGFP reporter mouse expresses eGFP in alpha cells (arrows). (B) Beta cells, marked by co-expression of UCN3 and mCherry, and delta cells (arrows) are GFP-negative. (**C**) S100b transcript is virtually undetectable, suggesting that eGFP expression is a useful artifact.
