# Supplementary material for: The transcriptional landscape of mouse beta cells compared to human beta cells reveals notable species differences in long non-coding RNA and protein-coding gene expression
Source: BMC Genomics. 2014 Jul 22;15(1):620. doi: 10.1186/1471-2164-15-620 (PMC4124169; doi:10.1186/1471-2164-15-620)
Supplement: Supplementary file 4 — Additional file 4: Is a side-by-side comparison of our alpha and beta cell transcriptomes with the beta cell transcriptomes derived from MIP-GFP + beta cells [20]. (PDF 277 KB) [file 12864_2014_6324_MOESM4_ESM.pdf]

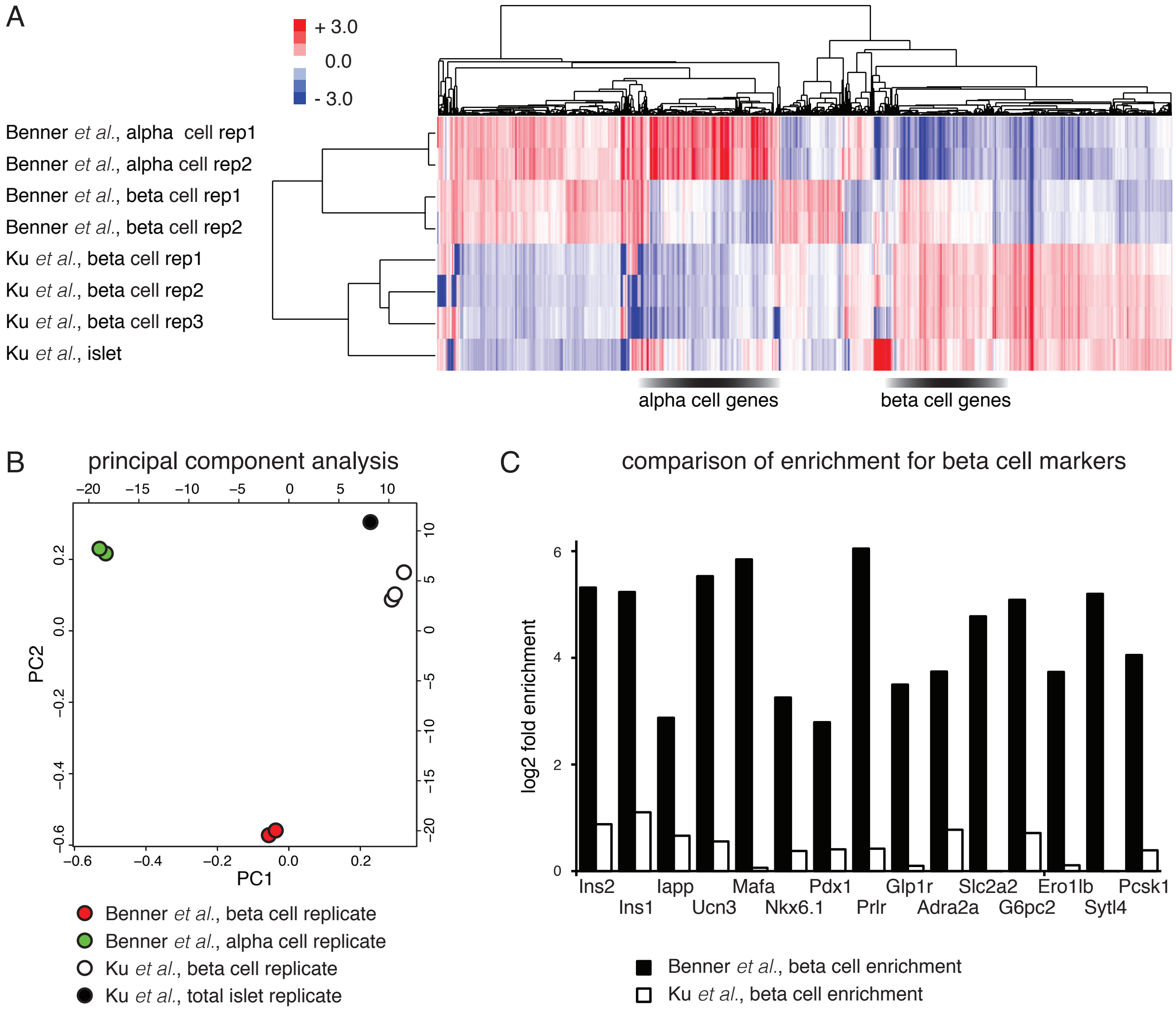

Additional data file 4: Comparison of our alpha and beta cell transcriptomes obtained from mIns1-H2b-mCherry x S100b-eGFP bitransgenic mice to the beta cell transcriptomes acquired by Ku et al., [1] from MIP-eGFP+ beta cells compared to whole islets. To enable a fair comparison, the raw data files representing FACS-purified MIP-GFP+ beta cells and total islet from [1] were downloaded and analyzed according to our analysis pipeline. Cluster analysis reveals a notably stronger contrast between our alpha and beta cells, then between the beta cell and total islet libraries used as contrast by in [1], particularly in the clusters representing alpha cell genes and beta cell genes (A). Principal component analysis also reflects the greater contrast between our beta and alpha cell libraries, while the contrast between purified beta cells and total islet libraries of [1] is decidedly smaller (B). This strong contrast between beta and alpha cells is reflected in the robust enrichment of established beta cell markers (C). In contrast, enrichment for these beta cell markers in MIP-GFP+ beta cells contrasted to total islet, as was done by [1], is predictably underwhelming, with several beta cell markers such as Mafa, Slc2a2 and Sytl4 not enriched in purified beta cells at all. Note the log2 scale in panel C.

1. Ku GM, Kim H, Vaughn IW, Hangauer MJ, Myung Oh C, German MS, McManus MT: Research resource: RNA-Seq reveals unique features of the pancreatic beta-cell transcriptome. Mol Endocrinol 2012, 26:1783-1792.
