# Supplementary material for: The transcriptional landscape of mouse beta cells compared to human beta cells reveals notable species differences in long non-coding RNA and protein-coding gene expression
Source: BMC Genomics. 2014 Jul 22;15(1):620. doi: 10.1186/1471-2164-15-620 (PMC4124169; doi:10.1186/1471-2164-15-620)
Supplement: Supplementary file 7 — Additional file 7: Is a figure containing single channel images for the immuno-fluorescent panels of Figure 4 . (PDF 2 MB) [file 12864_2014_6324_MOESM7_ESM.pdf]

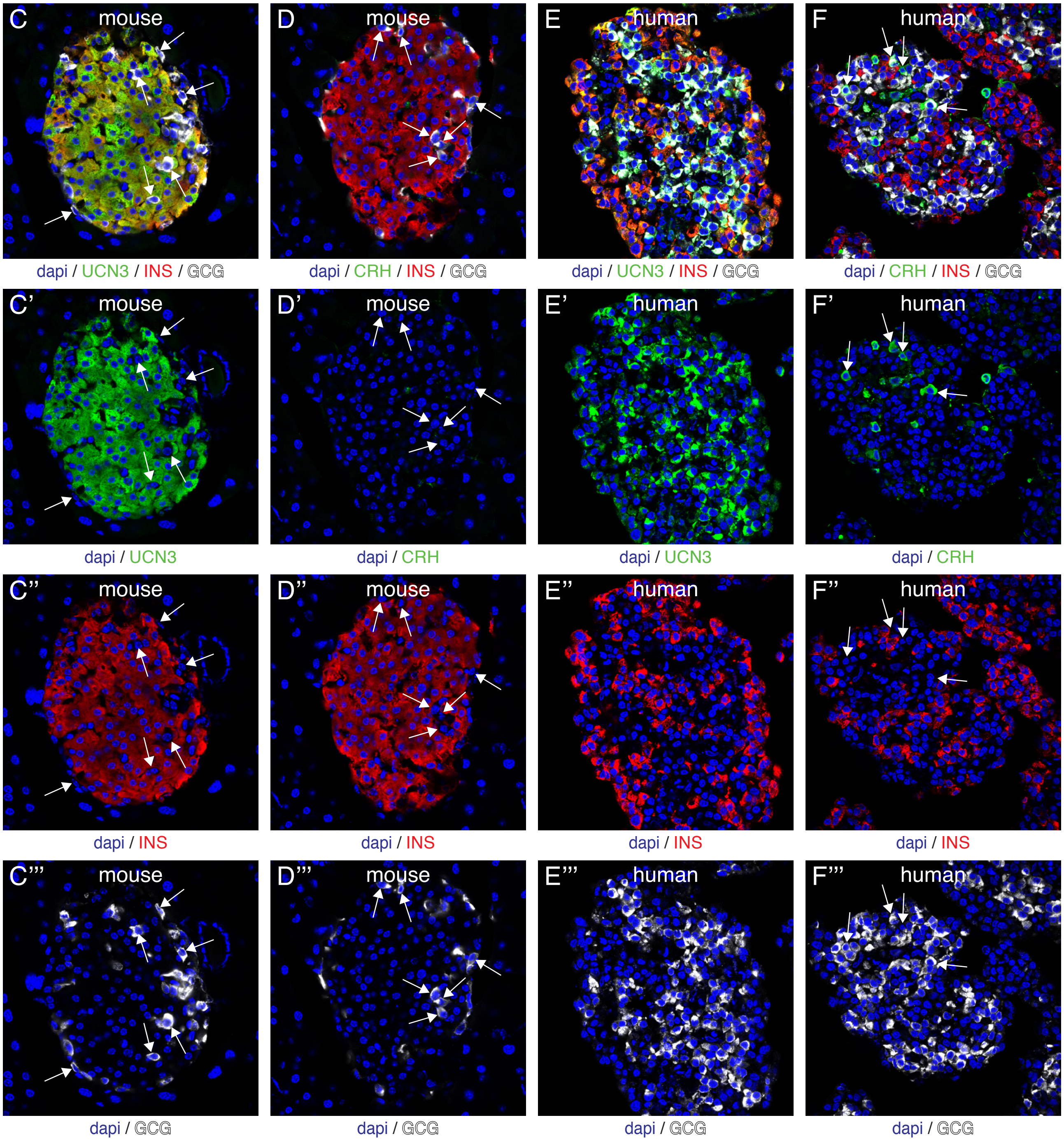

Additional data file 7: Validation of known differences in gene expression between mouse and human alpha and beta cells. Single channel panels of the immunofluorescence in Figure 5.
