# Supplementary material for: The transcriptional landscape of mouse beta cells compared to human beta cells reveals notable species differences in long non-coding RNA and protein-coding gene expression
Source: BMC Genomics. 2014 Jul 22;15(1):620. doi: 10.1186/1471-2164-15-620 (PMC4124169; doi:10.1186/1471-2164-15-620)
Supplement: Supplementary file 10 — Additional file 10: Is a side-by-side comparison of a standard curve of mouse and human IAPP as measured by ELISA, demonstrating 100% cross-reactivity. (PDF 121 KB) [file 12864_2014_6324_MOESM10_ESM.pdf]

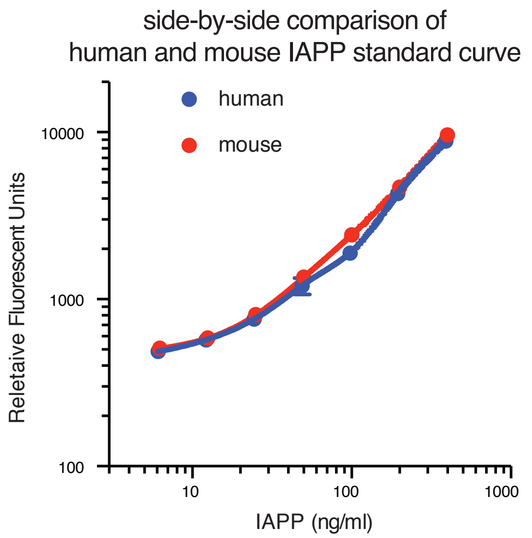

Additional data file 10: Standard curves for human and mouse IAPP demonstrate that IAPP from both is detected equally by the human IAPP elisa. Standard bars reflect standard errors across duplicates.
