# Supplementary material for: The transcriptional landscape of mouse beta cells compared to human beta cells reveals notable species differences in long non-coding RNA and protein-coding gene expression
Source: BMC Genomics. 2014 Jul 22;15(1):620. doi: 10.1186/1471-2164-15-620 (PMC4124169; doi:10.1186/1471-2164-15-620)
Supplement: Supplementary file 6 — Additional file 6: Is a figure highlighting the differential expression of genes important for glucose uptake and glycolysis, stimulus secretion coupling and insulin exocytosis in mouse beta and alpha cells. (PDF 536 KB) [file 12864_2014_6324_MOESM6_ESM.pdf]

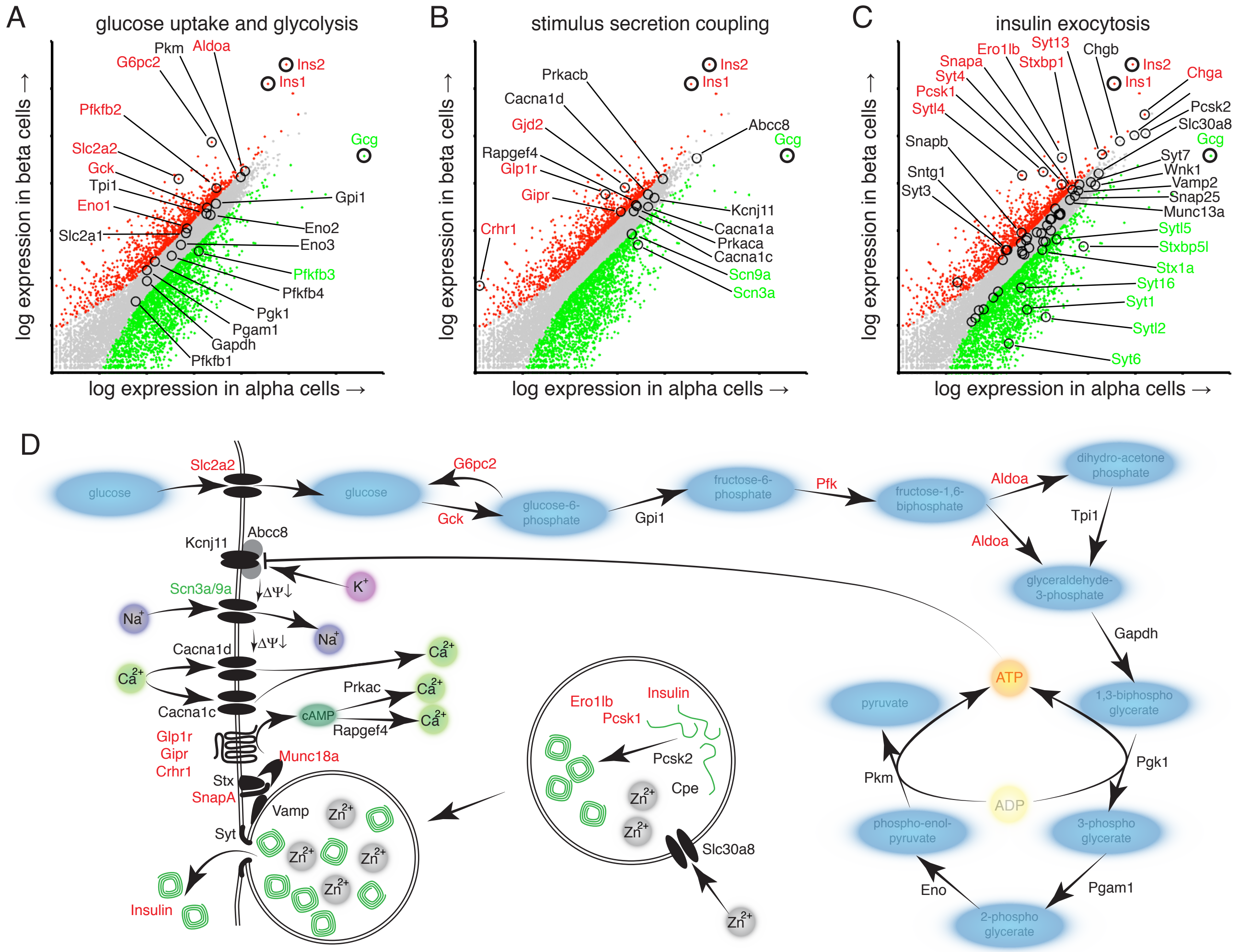

Additional data file 6: Stimulus-appropriate secretion of insulin constitutes the core function of the beta cell. We therefore looked at the genes required for glucose uptake and glycolysis (A), stimulus secretion coupling (B) and insulin exocytosis (C) - interconnected processes vital for the release of insulin dependent on ambient glucose concentration as summarized schematically in (D) and reviewed in detail elsewhere [1-3]. Among the most significantly enriched genes in our data that are involved in beta cell stimulus secretion coupling are the facilitated glucose transporter Slc2a2 (a.k.a. Glut2) and glucose-6-phosphate convertase 2 (G6pc2), which are selectively expressed in mouse beta cells [4, 5]. G6pc2, which regulates fasting glucose [6-8] and is an autoantigen for type 1 diabetes [9], is the 11th most abundantly expressed gene in mouse beta cells (Figure 3C). Ero 1-like beta (Ero1lb), involved in disulphide formation between the insulin amino acid chains, and proprotein convertase subtilisin/kexin type 1 (Pcsk1), specifically required for insulin but not glucagon processing, are highly enriched in beta cells as expected (Figure 3D). The most significantly enriched in mouse beta cells among the many proteins that coordinate docking and fusion of the secretory vesicle with the cell membrane is synaptotagmin-like 4 (Sytl4, a.k.a. granuphilin) (Figure 3E), which in mouse islets is required for granule docking to the plasma membrane while preventing vesicle fusion and insulin release [10, 11]. Finally, class B GPCRs such Glp1r, Gipr and Crhr1 that can potentiate glucose-stimulated insulin secretion [12, 13], are significantly enriched in beta cells.

1. Ashcroft FM, Rorsman P: Diabetes mellitus and the beta cell: the last ten years. Cell 2012, 148:1160-1171.
2. Eliasson L, Abdulkader F, Braun M, Galvanovskis J, Hoppa MB, Rorsman P: Novel aspects of the molecular mechanisms controlling insulin secretion. J Physiol 2008, 586:3313-3324.
3. Wang Z, Thurmond DC: Mechanisms of biphasic insulin-granule exocytosis - roles of the cytoskeleton, small GTPases and SNARE proteins. J Cell Sci 2009, 122:893-903.
4. Hutton JC, Eisenbarth GS: A pancreatic beta-cell-specific homolog of glucose-6-phosphatase emerges as a major target of cell-mediated autoimmunity in diabetes. Proc Natl Acad Sci U S A 2003, 100:8626-8628.
5. McCulloch LJ, van de Bunt M, Braun M, Frayn KN, Clark A, Gloyn AL: GLUT2 (SLC2A2) is not the principal glucose transporter in human pancreatic beta cells: implications for understanding genetic association signals at this locus. Mol Genet Metab 2011, 104:648-653.
6. Pound LD, Oeser JK, O'Brien TP, Wang Y, Faulman CJ, Dadi PK, Jacobson DA, Hutton JC, McGuinness OP, Shiota M, O'Brien RM: G6PC2: a negative regulator of basal glucose-stimulated insulin secretion. Diabetes 2013, 62:1547-1556.
7. Bouatia-Naji N, Rocheleau G, Van Lommel L, Lemaire K, Schuit F, Cavalcanti-Proenca C, Marchand M, Hartikainen AL, Sovio U, De Graeve F, et al: A polymorphism within the G6PC2 gene is associated with fasting plasma glucose levels. Science 2008, 320:1085-1088.
8. Chen WM, Erdos MR, Jackson AU, Saxena R, Sanna S, Silver KD, Timpson NJ, Hansen T, Orru M, Grazia Piras M, et al: Variations in the G6PC2/ABCB11 genomic region are associated with fasting glucose levels. J Clin Invest 2008, 118:2620-2628.
9. Lieberman SM, Evans AM, Han B, Takaki T, Vinnitskaya Y, Caldwell JA, Serreze DV, Shabanowitz J, Hunt DF, Nathenson SG, et al: Identification of the beta cell antigen targeted by a prevalent population of pathogenic CD8+ T cells in autoimmune diabetes. Proc Natl Acad Sci U S A 2003, 100:8384-8388.
10. Gomi H, Mizutani S, Kasai K, Itohara S, Izumi T: Granuphilin molecularly docks insulin granules to the fusion machinery. J Cell Biol 2005, 171:99-109.
11. Kato T, Shimano H, Yamamoto T, Yokoo T, Endo Y, Ishikawa M, Matsuzaka T, Nakagawa Y, Kumadaki S, Yahagi N, et al: Granuphilin is activated by SREBP-1c and involved in impaired insulin secretion in diabetic mice. Cell Metab 2006, 4:143-154.
12. Baggio LL, Drucker DJ: Biology of incretins: GLP-1 and GIP. Gastroenterology 2007, 132:2131-2157.
13. Huising MO, van der Meulen T, Vaughan JM, Matsumoto M, Donaldson CJ, Park H, Billestrup N, Vale WW: CRFR1 is expressed on pancreatic beta cells, promotes beta cell proliferation, and potentiates insulin secretion in a glucose-dependent manner. Proc Natl Acad Sci U S A 2010, 107:912-917.
